# Osteochondrosis, but not lameness, is more frequent among free-range pigs than confined herd-mates

**DOI:** 10.1186/s13028-015-0154-7

**Published:** 2015-09-29

**Authors:** Pernille Engelsen Etterlin, David A. Morrison, Julia Österberg, Bjørnar Ytrehus, Eva Heldmer, Stina Ekman

**Affiliations:** Section of Pathology, Department of Biomedical Sciences and Veterinary Public Health, Swedish University of Agricultural Sciences (SLU), 750 07 Uppsala, Sweden; Systematic Biology, EBC, Uppsala University, 752 36 Uppsala, Sweden; National Veterinary Institute (SVA), 751 89 Uppsala, Sweden; Terrestrial Ecology Department, Norwegian Institute for Nature Research (NINA), 7485 Trondheim, Norway; Swedish Animal Health Service (SvDHV), 464 32 Mellerud, Sweden

**Keywords:** Organic, Lameness, Housing, Gait scoring, Joints, Arthritis, *Erysipelothrix rhusiopathiae*, Osteochondrosis, Welfare

## Abstract

**Background:**

Organic pig production is expanding and amongst the objectives of organic farming are enhancing animal health and welfare. However, some studies have reported a higher prevalence of lameness and joint condemnation at slaughter in free-range/organic pigs than in conventionally raised pigs. Organic slaughter pigs have free-range housing in which indoor and outdoor access is compulsory, while in conventional farming the pigs are commonly confined to indoor pens. The present study evaluated the effects of free-range and confined housing on lameness prevalence in a herd of 106 finisher pigs, and whether osteochondrosis and *Erysipelothrix rhusiopathiae* associated arthritis influences these effects. We also evaluated the association between clinical lameness during the rearing period and joint condemnations at slaughter.

**Results:**

Seventy free-range and 36 confined housed fattener pigs were scored for their gait twice during the rearing period and 848 joints were evaluated post mortem. Osteochondrosis was more frequent among free-range than confined pigs (*P* < 0.05), and when present it was also more severe (*P* < 0.001). Pigs with more numerous and more severe osteochondral lesions had their gait affected more than did pigs with fewer such lesions (*P* < 0.05). Hence it was a paradox that we did not detect more lameness among the free-range pigs than the confined pigs. *E. rhusiopathiae* associated arthritis was not diagnosed. The association between gait remarks/clinical lameness and joint condemnations at slaughter was not significant.

**Conclusions:**

The results indicate that free-range housing may have both positive and negative effects on locomotory traits. Free-range pigs may be less clinically affected by osteochondrosis than are confined pigs. One explanation for this effect may be strengthening of joint supportive tissue and pain relief promoted by exercise. Visual gait scoring missed serious joint lesions that probably were harmful to the pigs, and should therefore not be used as a sole indicator of joint/leg health in welfare inspection of pigs. The association between gait scores and joint condemnation appeared to be poor. This study was limited to one herd, and so more and larger studies on the effects of free-range housing on lameness severity and osteochondrosis development in pigs are recommended.

**Electronic supplementary material:**

The online version of this article (doi:10.1186/s13028-015-0154-7) contains supplementary material, which is available to authorized users.

## Background

The ancestor of domestic pigs (*Sus scrofa domestica*) is the wild hog (*Sus scrofa ferus*) [1]. Wild hogs are exposed to predators [2] and hunting, and may roam over large areas [3]. Consequently, good locomotion is important for their survival, and the ability to run must be regarded as an important fitness (Darwinian) trait. Domestication of pigs has meant confinement and protection against predation, and commercial breeding has mainly focused on fast growth rate and carcass meat content/quality [4], thereby diminishing the selection pressure on locomotion traits.

However, interest in organic production of pork meat is increasing in several European Union countries [5, 6]. One of the goals in organic compared to conventional livestock husbandry is enhancing health and welfare [7]. The means for achieving these aims include letting the pigs range freely in large group housing with outdoor access, thus enabling them to express innate behavior. In Sweden the same pig breeds are used in both conventional and organic production [8]. Since these pigs have been bred for life in small indoor pens in which the area for movement and good locomotion is limited, their ability to cope in a more diverse environment may have diminished, and problems with lameness may potentially arise.

The effects that free-range housing has on lameness in organic finishing pigs have been insufficiently examined. The few studies comparing prevalences of lameness in free-range and conventional pigs have reported inconsistent results [9–12] and have not clarified the etiology of the lameness. Statistics gathered by the Swedish Animal Health Service show that between 1997 and 2014, joint condemnation rates in organic slaughter pigs were 2–5 times higher than in conventional slaughter pigs [13–15]. Badertscher and Schnider [9] also reported higher prevalence of joint condemnations at slaughter in free-ranging finisher pigs with outdoor access compared to conventional finishers, and such joint lesions may contribute to lameness.

Lameness has many causes [16], but among the most common in finisher pigs are joint lesions [17]. Several studies suggest that osteochondrosis (OC) [15, 18, 19] and erysipelas arthritis (ERA), caused by *Erysipelothrix rhusiopathiae* (ER) [20–22], are common in organic/free-range pigs. OC is caused by local ischemic chondronecrosis [23] leading to focal failure of endochondral ossification [24]. Osteochondrosis dissecans (OCD), with cracks from the necrotic cartilage through the articular surface and subsequent synovitis, is the most serious manifestation of OC [23]. ERA and OCD are considered to be two of the most painful causes of lameness in finishers [25], but their relative contribution to lameness in organic finishers has not been clarified.

The aim of this study was to examine the effect of free-range and confined housing on lameness in finishing pigs in relation to differences in prevalence of OC/OCD and/or ERA and/or joint condemnations. We hypothesized that: (1) free-range pigs would show more lameness than do confined pigs, which would be reflected in a difference in gait scores (evaluating locomotion); (2) gait score would relate to OC index; (3) gait score would relate to prevalence of ERA; and (4) pigs with poor gait scores at rearing would have their joints condemned at slaughter.

## Methods

The Gothenburg Ethical Committee on Animal Research (Dnr: C56/12) approved the use of the pigs in this study. The study population of 150 Hampshire × Yorkshire × Landrace pigs was also used in an earlier study [15]. At 11 weeks of age, 150 pigs born within the same week into one commercial organic breeding herd in Sweden were gathered in an enclosure, randomly selected and ID marked. The following week the pigs were moved to a finisher farm, where 50 pigs were distributed 5–7 in each of eight 12 m^2^ indoor pens with concrete floors. In another farmhouse, the remaining 100 pigs were divided into two 90 m^2^ group pens with deep straw bedding, with access to an outdoor concrete paddock as well as a common 50 m × 50 m enclosed pasture. All pigs were cared for and fed following EU regulations on organic farming [26] and according to weight following the SLU standard for feeding finisher pigs [27].

### Evaluation of locomotion (gait scoring)

Gait scoring to evaluate locomotion was performed once on each pig at 18 and 26 weeks of age. Each pig was singled out, and required to walk into a neighboring empty group pen (free-range pigs) or corridor (confined pigs), both with concrete floors without bedding. The pig’s gait was thereafter evaluated from the front, back and both side views. The pig was left to walk freely, but was approached if it stood still or lay down, to promote further walking. Gait was scored according to the scale shown in Table 1, a modified version of a four-point gait scale presented in the Welfare Quality^®^ reports [28]. Pigs were also observed for obvious injuries or signs of trauma to the legs or claws. As the pigs were housed separately, gait scoring without knowledge of the group allocation was not possible. Gait scoring was performed by the first author.

**Table 1:** Scores used to evaluate pigs’ gait

Scale	Gait	Criteria(s)
0	Normal	No visible gait deviation
1	Irregular	Tendency to abnormal stride length, or a slightly uneven weight bearing on one or more legs
2	Mild–moderate lameness	Obvious deviation in weight bearing on one or more legs, with clear difficulties walking
3	Severe lameness	No weight on the affected leg(s)/could not walk

### Slaughter and postmortem examination

The pigs were sent to slaughter between the ages of 25 and 29 weeks, when most pigs had reached a live weight of 95–110 kg. They were transported by truck and slaughtered at a commercial slaughterhouse situated 116 km from the farm. The right and left shoulder, elbow, stifle and hock joints of all pigs, including joints condemned at slaughter, were assessed for synovitis and OC as previously described [15]. All joints were disarticulated, and joints with synovial membrane or synovial fluid discoloration or an increase in synovial fluid volume were sampled at three synovial membrane sites for histological examination of inflammation (synovitis) and the presence of bacteria (Mayer’s hematoxylin and eosin and Gram stain). OC was scored on bone slabs using a six-point scale with 0 defined as no osteocondral lesions; 1, minor; 2, small; 3, moderate; 4, extensive; and 5 equivalent to an OCD lesion. The anatomical locations in which the articular surfaces were scored for OC are shown in Table 2.

**Table 2:** Locations in different joints examined for osteochondrosis

Shoulder	Distal scapula; glenoid cavity
	Proximal humerus; head of humerus
Elbow	Distal humerus; medial and lateral part of the condyle
	Proximal ulna
	Proximal radius
Stifle	Distal femur; medial and lateral oblique sulci and condyle, femoral trochlea
	Proximal tibia
	Proximal fibula
Hock	Distal tibia
	Distal fibula
	Proximal (medial and lateral trochlea) and distal talus
	Coracoid process and distal calcaneus

### Detection of *Erysipelothrix rhusiopathiae*

#### Serological examination for antibodies against *Erysipelothrix rhusiopathiae*

None of the pigs were vaccinated against ER. To get an overview of the herds’ exposure to ER, seroprevalence of ER was investigated. Blood samples without additives were collected from each pig at 11, 18 and 26 weeks of age. Serum was extracted and frozen at −20 °C until analyzed. Serum antibodies against ER were measured with a previously described indirect enzyme-linked immunosorbent assay (ELISA) [29, 30]. The cut-off absorbance value, based on sera from 83 specific pathogen free (SPF) pigs aged 10–12 weeks, was defined as O_450_ = 0.2 (mean + 3 SD + 0.1 for the 83 SPF pigs) [21, 30].

#### Immunohistochemistry

All joints with histologically confirmed synovitis were examined immunohistochemically for ER antigen. The primary antibody employed was a polyclonal antiserum against ER serotypes 1a, 1b and 2. This antibody, as well as a paraffin-embedded sample of a porcine lymph node positive for ER antigen, was kindly donated by Tanja Opriessnig [31]. The protocol was slightly modified (details provided in Additional file 1).

#### PCR

To further verify the results, a frozen synovial membrane sample from each joint with synovitis but no OCD was examined by a conventional ER-specific PCR assay. A kidney sample from a naturally ER-infected chicken was used as a positive control, and ultra-pure water and a sample of normal porcine synovial membrane were used as negative controls. DNA was extracted from the samples by the Qiagen DNeasy Blood and Tissue Kit^®^ (spin column protocol for purification of total DNA from animal tissues) according to the manufacturer’s instructions. Primers ER1 and ER2 [32] were used to detect a 937 bp fragment of ER as previously described [33]. The templates were analyzed at 1:1 and 1:10 concentrations, and reactions were duplicated. PCR products were visualized on 1.5 % agarose gel with SYBR^®^ Safe DNA gel stain (Invitrogen, Eugene, OR, USA).

### Statistical analysis

All descriptive and statistical analysis were performed using Minitab^®^ Version 16 (Minitab Inc, PA, USA) or Microsoft Excel for Mac 10.1.9. The larger of the left and right scores for each variable was used. Significance was defined as *P* ≤ 0.05 for all analyses.

Apart from calculating frequencies, percentages, means and standard deviations of the raw data, statistical differences between the two housing groups were analyzed with a multi-factorial ANOVA general linear model. For these ANOVAs housing group and sex were fixed factors, the interaction between these two factors was the fixed interaction effect, and slaughter weight was a covariate. The response variables were the gait scores at weeks 18 and 26 and the OC score-based “OC values” as defined in Table 3. The OC values were analyzed based on individual joints (shoulder, elbow, stifle, hock) and/or for the whole pig. Whole-pig results were calculated using the highest OC score recorded in any of the four joints in the analysis of “Highest OC”, “No OC” and “Highest OC 1, 2, 3, 4 or 5”, whereas “Sum of OC” used the overall sum of OC scores in all four examined joints.

**Table 3:** “OC values” used as ANOVA response variables

“Highest OC”	Highest OC score recorded in any location
“No OC”	Percentage of locations with no OC lesions (score 0)
“Highest OC 1, 2, 3, 4 or 5”	Percentage of locations at which OC scores 1, 2, 3, 4 or 5 (OCD), respectively, were the highest recorded
“Sum of OC”	Sum of all OC scores of all examined locations

*OC* osteochondrosis, *OCD* osteochondrosis dissecans

A second ANOVA model was used to examine the association between gait scores and OC, with the gait scores at week 26 as a response variable. This association was also analyzed at the joint and whole-pig level. The initial ANOVA model included: housing, sex, “OC value”, and their 2-factor interactions, with slaughter weight as a covariate. The interactions of “OC value” with sex and housing were not statistically significant, whereas a significant effect of slaughter weight was noted in some analyses. Therefore, the final ANOVA model analyzing the association between gait scores and OC included only: housing, sex, “OC value”, and the housing × sex interaction, with slaughter weight as a covariate.

Spearman rank correlations were calculated between joint condemnations (yes 1/no 0) in the whole pig and gait scores at week 26. A log-likelihood contingency test with Williams correction was used to examine the association between the binary variables joint condemnations (yes 1/no 0) in the whole pig and gait remark (yes 1/no 0).

## Results

Fourteen pigs were excluded: three free-range pigs were euthanized due to illness, three free-range and four confined pigs lost their ear tag IDs, three free-range pigs were slaughtered more than 1 month later than all of the other pigs, and one confined pig had missing slaughter data. Due to a misunderstanding, the farmer sent 30 (21 free-range, 9 confined), of the remaining 136 pigs to slaughter in week 25, i.e. 1 week prior to the final gait scoring and blood sampling. Consequently, these results are missing (missing at random [34]) for these 30 pigs, and they are excluded from all the data presented in this study. However, a separate paragraph has been included in which the results of the 136 pigs (whole pig level) are summarized.

Pigs with complete data sets included 25 castrates (barrows) and 45 gilts in the free-range group (70 total), and 16 castrates and 20 gilts in the confined group (36 total). In the free-range group, mean slaughter weight was 91.0 ± 4.4 kg for castrates and 91.4 ± 5.5 kg for gilts. In the confined group, mean slaughter weight was 99 ± 6.4 kg for castrates and 94.2 ± 8.5 kg for gilts.

### Gait scores

**Fig. 1:**
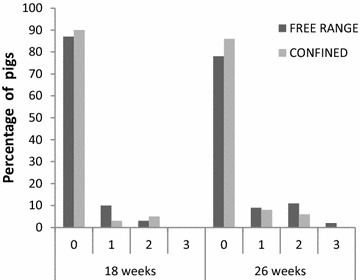
Prevalence of free-range and confined pigs with a gait remark at 18 and 26 weeks of age. The four scores on the x-axis are *0* normal gait, *1* irregular gait, *2* mild–moderately lame, *3* severely lame. There were no statistically significant differences between the two housing groups in the prevalence of pigs with a gait remark (score >0) at either sample time

The results of gait scoring for locomotion at 18 and 26 weeks, analyzed using the first ANOVA model, are shown in Fig. 1. At week 18, 9 % of gilts and 15 % of castrates received a remark about their poor gait, while at week 26 15 % of gilts (*P* = 0.291) and 24 % of castrates (*P* = 0.271) received a gait remark. The prevalence of pigs with a gait remark (i.e. score 1, 2, or 3) or clear lameness (gait score 2 and 3) did not significantly differ between groups and sexes at any sampling time. No pigs had obvious injuries to the legs, foot or claw at any of the gait scoring occasions. One free-range pig with severe lameness at week 26 was euthanized for ethical reasons the day after gait scoring and examined post-mortem for OC or ERA.

### Serum antibodies against *Erysipelothrix rhusiopathiae*

All pigs in both groups were ER seropositive with absorbance values >0.2 at all three sampling occasions.

### Synovitis, immunohistochemistry and PCR

Of the 848 joints examined, 74 displayed synovitis, and 66 (89 %) of these had OCD.

Histology revealed no bacteria, and immunohistochemistry did not detect ER antigens in synovial membrane. The remaining 8 joints with synovitis and no OCD examined with PCR were all negative. Histology, immunohistochemistry and PCR results were therefore in complete agreement that no joints with synovitis showed evidence of ER infections.

### Osteochondrosis and the effect of housing

**Fig. 2:**
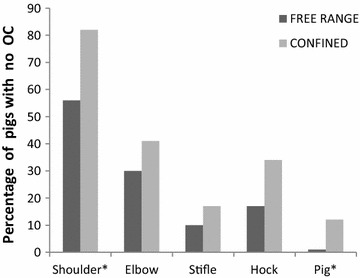
The estimated least-squares means for the prevalence (y axis) of joints/pigs (x axis) with no osteochondrosis (OC). Significant differences between the housing groups are designated as *P ≤ 0.05, **P ≤ 0.01, ***P ≤ 0.001 on the x axis. The confined pigs had significantly fewer lesions of OC in the shoulder joint and for the whole pig compared to the free-range pigs

**Fig. 3:**
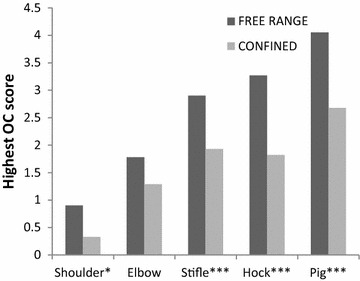
The estimated least-squares mean highest osteochondrosis scores (y axis) in the joints/pigs (x axis). Significant differences between the housing groups are designated as *P ≤ 0.05, **P ≤ 0.01, ***P ≤ 0.001 on the x axis. The OC scores ranged from score *0* (no osteocondral lesions present) to score *5* (severe OC lesion). The free-range pigs’ highest OC scores registered in the shoulder, stifle and the hock joint, and for the whole pig, were significantly higher than in the confined pigs

**Fig. 4:**
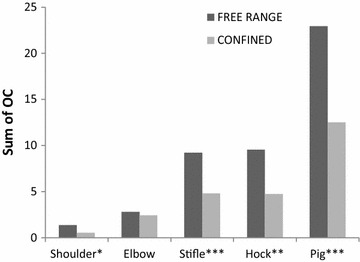
The estimated least-squares means “Sum of OC” (y axis) in the joints/pigs (x axis). Significant differences between the housing groups are designated as *P ≤ 0.05, **P ≤ 0.01, ***P ≤ 0.001 on the x axis. Free-range pigs had a significantly higher sum of OC in the shoulder, stifle and hock joints and for the whole pig compared to confined pigs

**Fig. 5:**
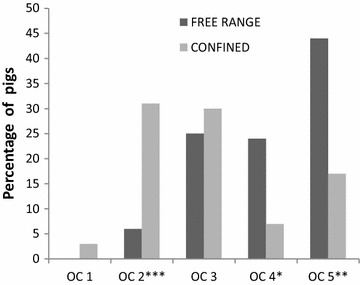
The estimated least-squares means for the prevalence of pigs (y axis) in each housing group with highest registered osteochondrosis (OC) scores of *1*, *2*, *3*, *4* or *5* (x axis). Significant differences between the housing groups are designated as *P ≤ 0.05, **P ≤ 0.01, ***P ≤ 0.001 on the x axis. The confined pigs significantly more often had OC 2 as the highest OC compared to the free-range pigs, which significantly more often had OC 4 or 5 as the highest value registered

Due to the unbalanced sample sizes across sex and housing groups, OC examination results (Figs. 2, 3, 4, 5) are presented as the least-squares means from the ANOVAs. OC was observed in at least one of four joints in 95 % (101) of all pigs, and OC prevalence differed significantly between the groups (free-range, 99 %; confined, 88 %). Significantly more numerous and more severe OC was found in many joints of the free-range pigs.

Castrates had a significantly higher “Sum of OC” in the stifle compared to gilts, but the differences between the sexes were otherwise not statistically significant. The interaction between group and sex was significant for “No OC” in the shoulder, and thus more confined castrates (0.97) than free-range castrates (0.50) had no OC lesions.

Slaughter weight had a statistically significant effect on “No OC”, “Highest OC” and the “Sum of OC” in the shoulder. The heavier the pig at slaughter, the more frequent and more severe OC lesions existed in the shoulder joints. The effect of sex, weight and group × sex interaction were not significant for other “OC values” in individual joints or the whole pig.

### Association between osteochondrosis and gait scores

Five percent of all pigs had “No OC” in any of the examined joints. Of the five pigs with “No OC”, four pigs had a normal gait and one had gait score 3 (severely lame). No joint lesions explaining the clinical findings in the latter pig were observed. A vertebral column or central nervous system disorder may be suspected.

The “Highest OC” scores and the “Sum of OC” in whole pigs with or without a gait remark at week 26 are shown in Table 4. Eleven of 37 pigs with an OCD in at least one location in any joint received a gait remark, and seven of these pigs had gait score 2 (mild–moderately lame). The second ANOVA model showed a statistically significant effect of the gait scores at week 26 for “Sum of OC” in the hock joint and “Sum of OC” in the whole pig. The higher the OC sum, the more likely the pig was to receive a gait remark. No other factors (sex, group, “OC values”), interaction effects (housing × sex), or covariates (slaughter weight) significantly affected gait scores at week 26.

**Table 4:** “OC values” in whole pigs with or without a gait remark at week 26

OC value	OC scores	Normal gait	Gait remark
Highest OC	Range	0–5	0–5
	Mean	3.5	4.0
	Standard deviation	1.3	1.4
Sum of OC	Range	0–49	0–50
	Mean	17.4	27.5
	Standard deviation	11.3	12.7
Number of pigs		86	20

*Normal gait* gait score 0, *Gait remark* gait score 1–3

### Joint condemnation and gait scores

Four joints from three pigs (all free-range) were condemned at slaughter. All of these had OCD and none had ER arthritis. One of these pigs received a gait remark at week 18, and all three received a gait remark at week 26. We found no significant association between joint condemnation and gait remark (yes/no) at week 26, but the correlation r = 0.35 between joint condemnation and gait score (0, 1, 2, 3) at week 26 was significant (*P* < 0.001).

### The effect of the 30 pigs that were missing at random

Twelve boars and 9 gilts in the free-range group and 6 boars and 3 gilts in the confined group were missing from the final data collection. Apart from significantly more confined (5 %) compared to free-range (<1 %) pigs having a maximum score of OC 1 in the group of 136 compared to 106 pigs, the whole-pig OC results were not significantly affected by this loss of 30 pigs. ER seroprevalence, ERA frequency and joint condemnations were not affected. Nevertheless, it is possible that the loss of statistical power prevented us from detecting some other potentially interesting biological effect(s).

## Discussion

This study, based on gait scoring of locomotion in 106 pigs and thorough examination of 848 joints, is the first to systematically investigate the effect of free-range versus confined housing on the prevalence and degree of lameness in organic finishing pigs, as well as the association of observed lameness with joint pathology and joint condemnation.

### Gait scoring and incidence of gait problems in free-range pigs

We used visual gait scoring, which has varying repeatability [35], because visual lameness evaluations are used in pig welfare inspections [36, 37] and are the only commonly used and practically feasible methods for assessing gait and lameness in a large number of pigs under farm conditions [38].

Our detailed study was limited to one herd (in Sweden). However the prevalence of lameness (gait score 2–3: 13 %) and gait remarks (score 1–3: 22 %) at 26 weeks in the free-range pigs is similar to that of finishers reported to have “problems in the locomotor apparatus” (10–15 %) in a Swiss survey of 116 organic pig farms [39], and the lameness prevalence (21 %) reported in nearly 700 Swedish free-ranging organic finishers [40]. Nevertheless, reports of the prevalence of lameness vary widely (1.6–21 %) at the end of the finisher period in free-ranging fatteners housed in organic/“animal friendly” systems with outdoor access [11, 12, 39–42]. Three of these studies also examined both confined and free-range fatteners and, consistent with our results, none of them found significant differences in prevalence of lameness between pigs in confined and free-range housing. However, another study concluded that free-range pigs were significantly more lame than conventionally housed pigs [9]. This large variation in published results shows the difficulty of determining the general prevalence of lameness in different populations of free-range and confined fatteners, but it may also be at least partly due to differences in the definition of lameness between studies [41].

We did not study the effect of specific factors associated with the housing conditions, but an important difference in housing between the free-range and confined pigs was the access to pasture. Nakano et al. [43] demonstrated that gait problems observed in pigs in indoor pens were not alleviated when they were moved to pasture. Sather [44] reported inconsistent effects on gait problems in association with pasture. Hence, more studies are needed to examine whether pasture promotes, protects or has a dual effect on lameness prevalence in free-ranging finisher pigs.

### The association between osteochondrosis and gait scores

Jørgensen et al. [45] suggested that a threshold (i.e. development of OCD and subsequent synovitis) must be exceeded before pigs show signs of lameness. Our results support this general idea, but not the specific suggestion that the threshold is development of an OCD lesion. We found that “Sum of OC” for the whole pig and in the hocks had a significant effect on gait score, whereas the severity of single OC lesions (“Highest OC”) did not. This indicates that, although OCD lesions contribute, it is only when pigs have a combination of many and severe lesions that pigs are likely to show clinical signs. Pigs unwillingly show signs of lameness [46], supporting the idea of a pain threshold in pigs and explaining why many OC lesions, but not single OCD lesions, seem necessary to generate visible lameness.

The connection between OC lesions and gait problems appears to be complex. It has been suggested that bilateral OC may mask lameness [45] and different studies on confined and group-housed indoor fatteners and sows have reported: no significant or an unclear association [45, 47], weak or assumed association [48, 49], or significant impact of OC on conformation/posture and/or gait kinematic [38, 50–52].

Paradoxically, although OC score differed significantly between our housing groups and was significantly associated with gait score, housing group did not contribute to this association, and gait score did not significantly differ between the groups. It seems unlikely that OCD lesions per se are less painful in free-range animals. Activity, however, is considered to improve biomechanical (skeletal, muscle, tendon) properties [53–56], and it relieves pain in humans with joint disease [57, 58]. Hence, because fatteners with outdoor access or in an enriched environment are more active and walk more than do confined pigs [59], their bone, muscle and tendon mechanical strength increase, leading to better joint support and improved biomechanics. An active pig may hence tolerate, up to a certain threshold, more severe OC lesions without clinical signs registered by gait scoring.

Consequently, our results imply that visual gait scoring is an insensitive tool for the evaluation of joint pathology in finishing pigs. Varying associations between visually assessed gait ability and OC have been reported, as mentioned above, but some studies also reported unclear associations between lameness and other types of lesions in the locomotor system such as leg or foot/claw lesions [28, 60]. This indicates the need for caution when using visual gait scores as evaluators of locomotion-associated welfare in pigs.

An essential component of welfare in organic production is to let pigs range freely. More research is therefore needed to understand whether it is more exercise, or this in combination with other housing factors, that promotes OC development [15], as well as a possible better ability to cope with OC in free-range pigs.

### Joint condemnation and gait scores

A previous study [40] on organic fatteners reported no/weak association between joint condemnations and lameness in fatteners, consistent with our study. The weak (but significant) correlation between gait scores and joint condemnations indicates that pigs in which joints are condemned tend to have higher gait scores. However, only a few joints were condemned, and so the statistical analyses have low power. Nevertheless, abattoir inspection of joints does not recognize the large majority of severe joint lesions [15], which is probably an important explanation for the lack of correlation between pigs with a gait remark and pigs with a joint condemned.

### *Erysipelothrix rhusiopathiae* seroprevalence and arthritis

Although all pigs in our study developed an antibody response against ER, no ERA cases were recorded, hence we could not test any possible association between ERA and lameness. ERA may be a less common cause of joint lesions in pigs than is OC/OCD. An earlier study, in which 70 % of joints of organic fatteners condemned at slaughter had OCD and 4 % had ERA [18], supports these findings. The importance of ERA as a cause of joint lesions and lameness in conventional pigs varies [17, 61]. Previous studies have suggested that ERA is common in Swedish organic fatteners [13, 20, 21]. These conclusions have largely been based on associations between ER seroprevalence and condemnations of unopened joints at slaughter in free-ranging organic finisher pigs. In none of these studies were the joints opened and the exact pathology evaluated, which is essential for the evaluation of ERA prevalence.

## Conclusions

In this study we found that a combination of many and severe OC lesions is significantly associated with irregular gait and lameness. However, although free-range pigs displayed significantly more numerous and more severe OC lesions than did confined pigs, housing group did not have a significant impact on gait scores evaluating locomotion. Hence, our hypothesis of more lameness in free-range than in confined pigs was not supported, indicating that some mechanism allowed free-range pigs to be less clinically affected by OC lesions. This also implies that lameness evaluation is unreliable as a means to detect joint pathology in pigs. No pigs developed ERA. Moreover, the association between gait problems and joint condemnations was poor. The over-representation of OC lesions in the free-range pigs was an important health issue and, as all pathology is considered to affect welfare [62], this must subsequently be considered as having been detrimental to the welfare of the free-range pigs. This study was limited to one herd, and thus the results should be validated in more free-range herds, perhaps with different genetic origins, along with more research on the effect of pasture and free-range activity on the development of OC and lameness.

## Additional file

10.1186/s13028-015-0154-7 Immunohistochemical protocol for detection of Erysipelothrix rhusiopathiae.

## Abbreviations

ER: *Erysipelothrix rhusiopathiae*
ERA: erysipelas arthritis
OC: osteochondrosis
OCD: osteochondrosis dissecans
