# Supplementary material for: Osteochondrosis, but not lameness, is more frequent among free-range pigs than confined herd-mates
Source: Acta Vet Scand. 2015 Sep 29;57:63. doi: 10.1186/s13028-015-0154-7 (PMC4587880; doi:10.1186/s13028-015-0154-7)
Supplement: Supplementary file 1 — 10.1186/s13028-015-0154-7 Immunohistochemical protocol for detection of Erysipelothrix rhusiopathiae. [file 13028_2015_154_MOESM1_ESM.docx]

Additional file 1

*Immunohistochemistry*

Slides of formalin-fixed paraffin-embedded synovial membrane sections were heated to 60°C for 30 minutes, dewaxed, dehydrated, incubated in citrate buffer pH 6 for 20 minutes at 92°C for antigen demasking, and rinsed in phosphate buffered saline (PBS). Endogenous peroxidase activity was quenched using 0.3% H_2_O_2_ in distilled water followed by rinsing in PBS. The slides were then incubated with the primary antisera, diluted 1:1500 in PBS, for 1 hour at room temperature. Further processing was performed using a LSAB kit (DAKO, Glostrup, Denmark) according to the manufacturer’s instructions. Sections from a porcine lymph node positive for ER antigen were used as positive controls, and as a negative control the ER antibody was omitted and non-immune rabbit IgG applied at the same concentration. The staining location (tissue and intracellular / extracellular) and density (minor, moderate, intense) were recorded, with non-staining considered negative for ER.
